# Meteorological conditions hardly influence measurement strategy and measured respirable dust and quartz concentrations in the industrial minerals sector

**DOI:** 10.1093/annweh/wxaf060

**Published:** 2025-10-06

**Authors:** Nicola Blagrove-Hall, Remko Houba, Alonso Bussalleu, Hans Kromhout

**Affiliations:** Institute for Risk Assessment Sciences, Utrecht University, PO Box 80178, 3508 TD Utrecht, The Netherlands; The Netherlands Expertise Centre for Occupational Respiratory Disorders (NECORD), Utrecht, The Netherlands; Department of Epidemiology, Swiss Tropical and Public Health Institute, Kreuzstrasse 2, 4123 Allschwil, Switzerland; Institute for Risk Assessment Sciences, Utrecht University, PO Box 80178, 3508 TD Utrecht, The Netherlands

**Keywords:** exposure modelling, large exposure database, meteorological conditions, mineral mining, respirable respirable dust, respirable quartz

## Abstract

**Background:**

Respirable dust and quartz are important occupational hazards, yet the effects of meteorological conditions on these concentrations remain poorly understood and predominantly theoretical.

**Objective:**

Using respirable dust and quartz data from the Industrial Minerals Association Europe Dust Monitoring Programme (IMA-DMP) and outdoor meteorological data from the ERA5-LAND hourly land data, we aimed first to determine whether meteorological conditions had an impact on the sampling strategy for these exposures and, second, to describe the association between outdoor meteorological conditions and respirable dust and quartz concentrations.

**Methods:**

We linked the exposure data for 153 sites across Europe and outdoor meteorological data by date and IMA site location. We used descriptive statistics to compare the meteorological conditions (temperature, precipitation, and wind speed) on measurement and non-measurement days. A linear mixed-effects model was used to investigate the relationship between meteorological variables and respirable dust and quartz concentrations. The model includes adjustments for period-specific time trends, minerals produced, job site, and job function.

**Results:**

Meteorological conditions on measurement and non-measurement days were similar. We estimate a 2.3% and 5.9% increase in dust and quartz concentrations for every 10 °C increase in temperature. A 10-fold increase in precipitation is estimated to reduce dust and quartz concentrations by −2.6% and −3.1%, respectively. A 10-fold increase in wind speed is estimated to reduce quartz concentrations by −9.0%, and this association was not statistically significant for dust. Temperature had the strongest effect on personal concentrations, followed by wind speed. Associations were generally stronger for respirable quartz than respirable dust.

**Conclusions:**

Within the IMA-DMP, meteorological conditions did not affect the measurement strategy for dust and quartz and had a small effect on concentrations measured at 153 sites across Europe. Thus, non-random, biased sampling schemes would result in a slight (<10%) overestimation or underestimation of long-term respirable dust and quartz concentration depending on the meteorological conditions, justifying the collection of meteorological data during sampling.

**What’s Important About This Paper?**

The relationship between respirable dust and quartz concentration and meteorological variables in an occupational setting was primarily theoretical until now. Using data from an industry monitoring program, this study found no bias in measurement strategy due to weather conditions. However, meteorological variables explain some variability in dust and quartz concentrations; therefore, weather data should be collected during sampling campaigns. The results contribute to our understanding of how workers’ exposures will be affected by global climate change.

**Highlights**

▪ First detailed study of patterns between dust and quartz exposure and meteorology.
▪ No meteorological-related biases in the sampling strategy for dust and quartz.
▪ Only sampling at warmer outdoor temperatures will lead to overestimated exposures.
▪ Only sampling at higher outdoor wind speed will lead to underestimated exposures.
▪ Precipitation is unlikely to affect estimated exposures.

## Introduction

Over the past few decades, silica exposure levels and rates of silicosis decreased across Europe; however, respirable crystalline silica remains a priority hazardous substance in the workplace due to its prevalence and new probable high exposure levels in artificial stone applications (Peters et al. 2012; Hoy *et al.* 2018, Hoy 2022).

Among other determinants of respirable dust and quartz exposures such as tasks performed, tools used and control measures present, various studies suggested that meteorological conditions may impact the respirable particulate matter and personal exposures and must therefore be considered when assessing ambient air exposures and personal occupational exposures (Moschandreas and Saksena 2002; Swanepoel *et al.* 2018; Duarte *et al.* 2022). A study carried out at residential and mine sites around coal projects, investigating the relationship between respirable PM (<4 μm) among other size fractions and meteorological parameters, found a weak negative correlation (Pearson correlation coefficient—*R*) between PM (<4 μm) and temperature (*R* ~ −0.5), and PM and wind speed (*R* ~ −0.4) (Sahu *et al.* 2018). The sparse literature available suggests that meteorological conditions might play a key role in the temporal variability in respirable dust and quartz concentration at work sites and should be included in the measurement strategy. It was reported that within the IMA-DMP database, 76% of the measurement days were dry days and 72% were not windy days, however, actual meteorological conditions however, self-reported yes–no variables were used to arrive at these estimates (Zilaout *et al.* 2017). It is currently unknown in the literature whether outdoor meteorological conditions (temperature, wind speed, and precipitation) impact the decision of the occupational hygienist to conduct sampling and whether meteorological conditions is associated with personal respirable dust and quartz concentration in the industrial mineral sector.

In this study, our 2 main objectives were first to compare outdoor temperature, wind speed, and precipitation between measurement days and non-measurement days using descriptive statistics and, second, to describe and compare relationships between these meteorological variables on measurement days and measured respirable dust and quartz concentrations using descriptive statistics, simple spline plots, and a linear mixed-effects model. These objectives were carried out using reliable estimates of outdoor site level meteorological data from ERA5-Land reanalysis database (Muñoz-Sabater 2019) and exposure data available within the IMA-DMP database.

## Materials and methods

### Database preparation

Industrial Minerals Association (IMA-Europe) includes European producers of andalusite, bentonite, cristobalite, diatomite, feldspar, kaolin, clays, and quartz. The Industrial Minerals Association Dust Monitoring Program (IMA-DMP) has been running for over 2 decades. Data collection started in 1999/2000 and has been managed collaboratively by the Netherlands Expertise Centre for Occupational Respiratory Disorders and the Utrecht University Institute of Risk Assessment Science since 2006. As of August 2022, the IMA-DMP consisted of 39 191 respirable dust measurements and 34 964 respirable quartz measurements. Respirable dust concentrations were analysed using gravimetric analysis and respirable quartz using either X-ray diffraction or infrared spectroscopy. As previously described, multiple samplers were used for the collection of respirable dust and quartz; therefore, adjustments were made to account for differences in sampler efficiencies and values below the limit of detection (LoD) were imputed using multiple imputation with a maximum likelihood estimation method (Zilaout *et al.* 2020).

**Fig. 1.**
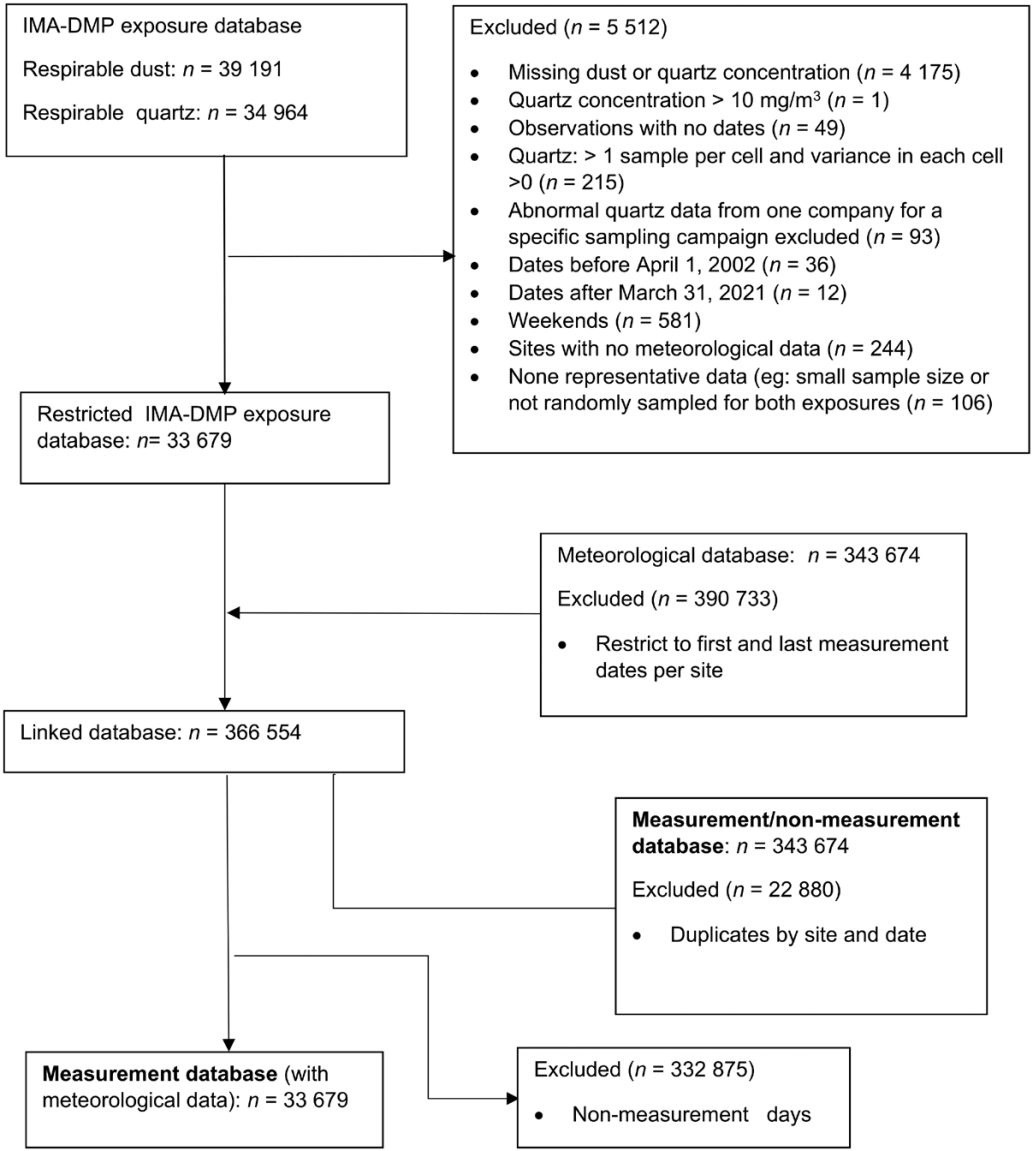
Database management flowchart showing decision steps used to arrive at the analysis databases used in this study.

The IMA-DMP exposure database was restricted to the measurement period 1 April 2002 (campaign 4) to 31 March 2021 (Campaign 41), and data were sparse prior to and after this period. The database was further restricted to representative data collected on weekdays (Monday to Friday) for which both respirable dust and quartz concentrations were measured and for which meteorological data were available (Fig. 1). The resulting database consisted of 33 679 simultaneously collected respirable dust and quartz measurements that were collected at 153 industrial sites from 30 companies across 22 countries for 7 mineral types and 12 job functions. First and last participation dates per site within the study period were flagged and these dates were used as the cut-points for the meteorological data. Verified site addresses were geocoded using a Google map API and the mutate geocode function in R. Randomly selected addresses were reverse geocoded for cross validation.

Land hourly meteorological data were extracted from ERA5-LAND the European Centre for Medium Range Weather Forecast (ECMWF) Weather Reanalysis of Global Climate version 5 (Muñoz-Sabater 2019; Blanco et al. 2023; Gomis-Cebolla *et al.* 2023). This is a global deterministic model calibrated with real data produced at 9 km (0.10 × 0.10) hourly resolution from 1950 to present. Hourly data were extracted from the ECMWF data portal for the point coordinates of IMA-DMP sites and gaps along coastlines in the ERA5-LAND data were filled using bilinear interpolation. For each site, hourly meteorological data were averaged over 24 h to provide daily, temperature (2 m above surface), total precipitation, eastward, and northward wind components (10 m above surface). Temperature in °K was converted to ^°^C, total precipitation to mm, and eastward and northward wind components were used to calculate windspeed (m/s) (Menochet, 2024).

The meteorological and the restricted IMA-DMP exposure databases were linked by site and date to create the 2 databases used for this study. The first analysis database, the measurement/non-measurement database (*n* = 343 674), was created by deleting duplicate observations per date-site and was used to describe and compare meteorological conditions on measurement and non-measurement days. The second database, the measurement database (*n* = 33 679), was created by deleting observations with no measurement data, and identical meteorological data were assigned when multiple measurements were taken per date-site. This measurement database was used to describe, compare and model relationships between meteorological conditions (temperature, precipitation, and windspeed) and respirable dust and quartz concentration. Zero values for precipitation (0.3%) were substituted for half the lowest value within the database (2.62 × 10⁻⁷ mm). There were no zero values for wind speed. Precipitation and wind speed were log_10_-transformed.

### Statistical analysis

The meteorological variables for measurement and non-measurement days were described using arithmetic mean (AM), standard deviation, median, and the interquartile range (IQR); associations among these variables were assessed using Spearman rank correlation. Respirable dust and quartz concentrations were summarized as AM, geometric mean (GM), and geometric standard deviation (GSD).

Simple spline plots were used to investigate patterns between meteorological variables and respirable dust and quartz concentration. We used identical linear mixed-effects model to evaluate the associations between meteorological conditions and respirable dust and quartz concentrations, enabling a direct comparison of these association. Apriori features of our mixed model included the following fixed effects; temperature, log_10_ transformed precipitation and wind speed, overall long-term and period-specific time trends, and random effects; and mineral, site within mineral, and job function within site (Table S1 for job function description). The interaction term between overall long-term time trend (1 April 2002 to 31 March 2021) and period was included in the model to account for variations in period-specific temporal trends. Period-specific time trends were defined as: early-period (1 April 2002 to 31 October 2008), middle-period (1 November 2008 to 31 October 2012), and recent-period (1 November 2012 to 31 March 2021) (Zilaout *et al.* 2020). Region was not included because it was the aggregate of site which was already included in the final model. Exposure variability and model fit were assessed using the Akaike information criterion (AIC), and the model with the best fit (lowest AIC) is presented. To account for differences in the dispersion characteristic of each meteorological variable, the IQR was used to calculate the robust semi-standardisation beta coefficients reported.

To account for any biases in exposure estimates due to the increasing number of non-detects particularly in the quartz dataset, a sensitivity analysis was performed which included modelling only exposures that were above the LoD. Sub-group modelling analyses were also performed, classifying exposures by temperature with a cut point of 6 °C and by region. Low temperature were defined as ≤6 °C and high temperature > 6 °C, whereas regions were defined by countries; north (Finland, Norway, and Sweden), west (Denmark, Belgium, France, Germany, Netherlands, Switzerland, and the United kingdom), east (Austria, Bosnia, Czech Republic, Hungary, Poland, Russia, Slovakia, and Ukraine), and south (Greece, Italy, Portugal, Spain, and Turkey). The data were analysed using SAS software (version 9.4 Copyright 2023 by SAS Institute Inc., Cary, NC, USA) and through a standardized R script (R version R 4.2.3 [March 2023]), including the following packages: “tidyverse, ggmap, openair.”

## Results

The IMA-DMP dataset of simultaneously collected respirable dust and quartz samples from 1 April 2002 to 31 March 2021 showed small meteorological differences between measurement and non-measurement days. The measurement/non-measurement day database consisted of 343 674 unique site-date combinations spanning 4908 calendar weekdays (potential measurement days). Of these 343 674 unique site-date combinations, dust and quartz measurements were collected on 10 799 (3%), corresponding to 3986 calendar weekdays. On these 3986 measurement days, at least 1 sample was collected at one site. On average, measurement days were 1 °C warmer, had 0.1-mm less total precipitation (therefore slightly drier), and had 0.3-m/s lower wind speed compared with non-measurement days (Table 1). Weather variables were weakly correlated: temperature and wind speed had a negative Spearman rank correlation (ρ = −0.28), precipitation and wind speed were positively correlated (ρ = 0.22), and temperature and precipitation were negatively correlated (ρ = −0.10). On average, 3.1 (range 1–40) exposure measurements were collected per measurement day per site, with an average sampling duration of 7 h.

**Table 1.** Summary of meteorological conditions on measurement (*n* = 10 799) and nonmeasurement days (*n* = 332 875).

	Day	AM^a^	Std Dev^b^	Median	IQR^c^	Minimum	Maximum
Temperature (°C)	Measurement	12.1	7.77	12.4	11.0	-22.9	31.6
	Non-measurement	11.0	7.31	11.1	10.3	-29.2	32.9
Precipitation (mm)	Measurement	1.13	2.21	0.28	1.19	0.00	53.9
	Non-measurement	1.19	2.20	0.32	1.32	0.00	60.0
Wind speed (m/s)	Measurement	2.44	1.68	2.13	2.25	0.02	12.6
	Non-measurement	2.71	1.73	2.40	2.28	0.00	17.0

a Arithmetic Mean, ^b^ Standard Deviation, ^c^ Inter-Quartile Range.

Sample collection frequency was inconsistent throughout the year and varied over time by region. Sampling occurred more frequently in March, June, and September, while it was least frequent in April (*n* = 1483). More samples were collected during the summer campaigns (20 326; 60%) than in the winter campaigns (13 353; 40%) (Fig. S1). Most exposure data were collected in the more recent-period (53% after October 2012), and the remaining was evenly distributed between the middle-period (24%) and the early-period (23% before November 2008). Not all countries or regions participated in the IMA-DMP from the start. In fact, participation increased over time, particularly from southern European countries, and ~10 yr into the program, eastern countries also began participating (Fig. S2).

Exposure concentrations varied moderately by mineral type, job function, and region, yet differences in meteorological conditions were most evident between regions (Table 2). Sampling in companies producing silica accounted for 60% of the exposure database. This mineral group had the lowest respirable dust concentration (GM = 0.10 mg/m^3^), and second lowest respirable quartz concentration (GM = 8.72 µg/m^3^) compared with other minerals produced. The least frequent exposure sampling occurred in bentonite mines (<1%), where some of the highest respirable dust concentrations were recorded (GM = 0.32 mg/m^3^); however, respirable quartz concentrations were relatively low (GM = 8.59 µg/m^3^). The highest respirable quartz concentrations was found in the mixed mineral group (GM = 17.1 µg/m^3^), which accounted for 10% of the exposure database. Among job function, bagging operators (12%) and plastification workers (<1%) had the highest respirable dust concentrations (GM = 0.28 and 0.25 mg/m^3^, respectively) and respirable quartz concentrations (GM = 35.2 and 21.4 µg/m^3^, respectively). Foremen (10%) and quarry operators (12%) showed lower respirable dust concentrations (GM = 0.08 mg/m^3^) and respirable quartz concentrations (GM = 5.16 and 4.84 µg/m^3^, respectively).

**Table 2.** A summary of respirable dust (mg/m^3^) and quartz (µg/m^3^) concentrations and meteorological conditions by mineral type, job function, and region (*n* = 33 679).

		Respirable Dust(mg/m^3^)			Respirable Quartz(µg/m^3^)			Median meteorological data measurement days			
	N^a^	AM^b^	GM^c^	GSD^d^	AM	GM	GSD	N^e^	T^f^ (^0^C)	P^g^ (mm)	WS^h^ (m/s)
**Overall**	33679	0.41	0.14	3.71	34.0	9.46	4.81	10799	12.4	0.28	2.13
**Mineral**											
Bentonite	92	0.51	0.32	2.68	16.4	8.59	2.77	54	12.8	0.62	2.48
Clay	4726	0.37	0.16	3.57	39.7	14.6	4.36	1085	12.6	0.22	2.26
Feldspar	1308	0.86	0.26	4.35	38.5	12.3	4.77	627	13.3	0.16	1.59
Kaolin	1314	0.63	0.17	4.44	19.6	5.96	3.69	355	12.0	0.45	3.30
Mixed minerals	3442	0.58	0.23	3.47	69.6	17.1	5.41	1212	13.3	0.35	1.88
Other minerals	3129	1.02	0.25	4.49	17.9	4.66	4.75	1171	11.2	0.25	2.70
Silica	19668	0.25	0.10	3.28	29.9	8.72	4.59	6295	12.4	0.29	2.06
**Job function**											
Bagging operator	4058	0.66	0.25	3.51	63.5	21.4	4.62	1264	11.5	0.27	2.26
Crusher operator	692	0.55	0.17	4.24	29.3	8.97	5.65	304	13.8	0.22	1.35
Dry process operator	3548	0.55	0.19	3.66	50.2	14.6	4.64	1349	12.2	0.23	2.13
Foreman	3290	0.21	0.08	3.22	15.1	5.16	4.30	388	12.7	0.27	1.76
Laboratory worker	2714	0.20	0.10	3.10	19.6	8.13	3.94	1520	12.8	0.29	2.19
Maintenance worker	4653	0.68	0.19	3.79	43.3	10.9	4.79	611	11.6	0.42	2.39
Miller operator	1476	0.51	0.20	3.42	51.8	18.3	4.37	621	11.8	0.35	2.27
Multi skilled worker	1368	0.39	0.14	3.81	33.6	7.97	5.18	889	12.9	0.24	1.88
Plastification worker	190	0.44	0.28	2.79	71.6	35.2	3.46	163	9.91	0.25	1.98
Quarry operator	3939	0.19	0.08	3.33	14.7	4.84	4.19	1911	12.4	0.27	2.18
Transport worker	5328	0.30	0.12	3.53	26.5	7.72	4.46	1005	13.4	0.23	1.97
Wet process operator	2423	0.27	0.11	3.44	21.8	8.30	3.99	774	12.0	0.33	2.28
**Region**											
East	2131	0.70	0.11	4.91	25.8	6.18	5.15	775	14.4	0.26	2.37
West	19006	0.42	0.13	3.99	39.3	9.73	5.14	5158	10.7	0.40	2.82
North	1896	0.58	0.21	3.74	49.8	17.4	4.45	1034	7.26	0.42	2.23
South	10646	0.31	0.15	2.92	23.7	8.78	4.07	3832	16.6	0.14	1.16

a Number of measurements, ^b^Arithmetic Mean. ^c^Geometric Mean, ^d^Geometric Standard Deviation, ^e^Number of measurement days, ^f^Temperature, ^g^ Precipitation, ^h^ Wind Speed.

Differences were observed in precipitation between mineral types—feldspar (0.16 mm) compared with bentonite (0.62 mm); however, no other notable differences in meteorological conditions were found between mineral types or job functions.

Moderate regional differences in respirable dust and quartz concentrations were also observed. The northern region (6%) had the highest respirable dust and quartz concentrations (GM = 0.21 mg/m^3^ and 17.4 µg/m^3^, respectively), accompanied by the lowest median temperature and the highest precipitation levels. In contrast, the eastern region (6%) had the lowest respirable dust and quartz concentrations (GM = 0.11 mg/m³ and 6.81 µg/m³, respectively) and average meteorological conditions. However, these regions accounted for only a small fraction of the exposure database. Most measurements were taken in the western region (56%, GM [dust] = 0.13 mg/m³ and GM [quartz] = 9.73 µg/m³) and the southern region (32%, GM [dust] = 0.15 mg/m³ and GM [quartz] = 8.78 µg/m³), where exposure levels were moderate. However, notable differences in meteorological conditions were seen between these regions: the western region was cooler, with higher precipitation and wind speed compared with the southern region.

**Fig. 2.**
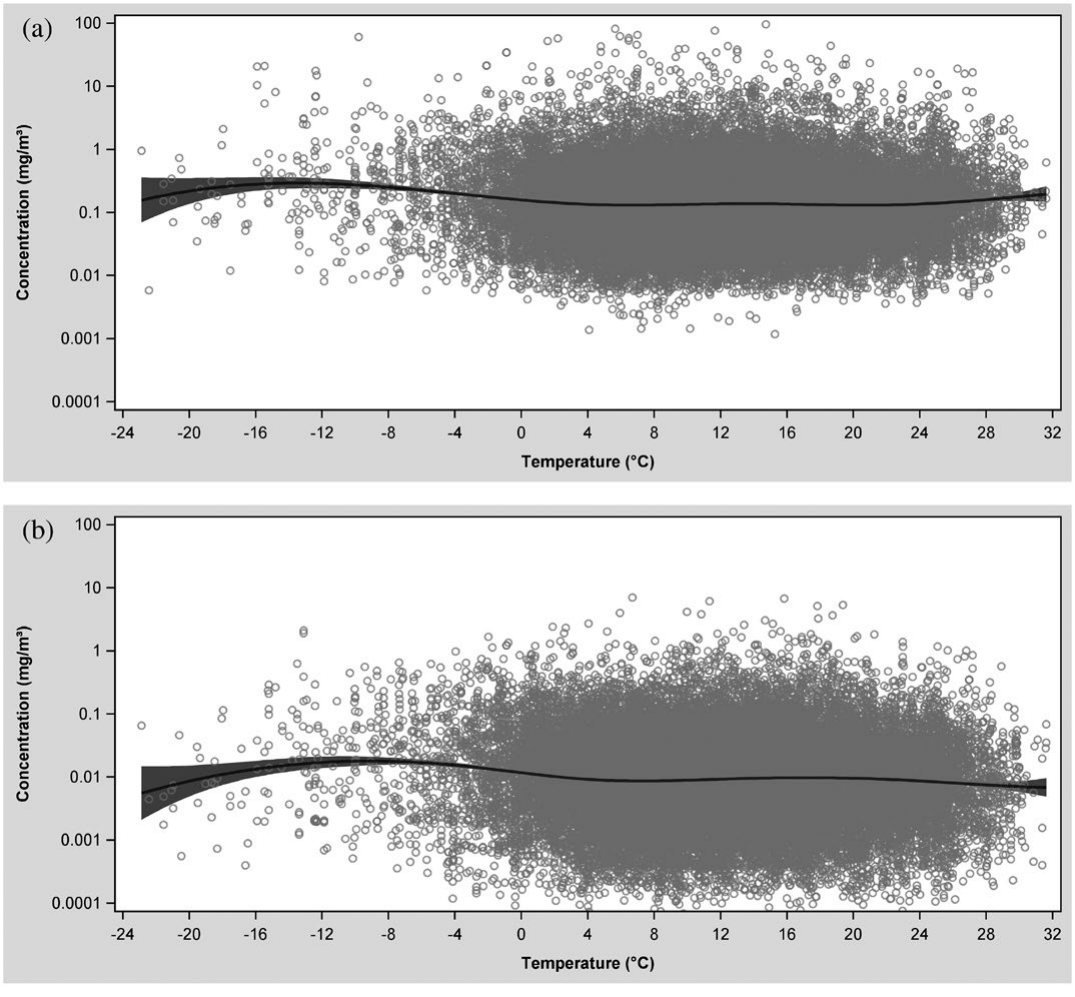
Spline plot of respirable dust (a) and quartz (b) concentrations in mg/m^3^ versus outdoor temperature in ^°^C.

**Fig. 3.**
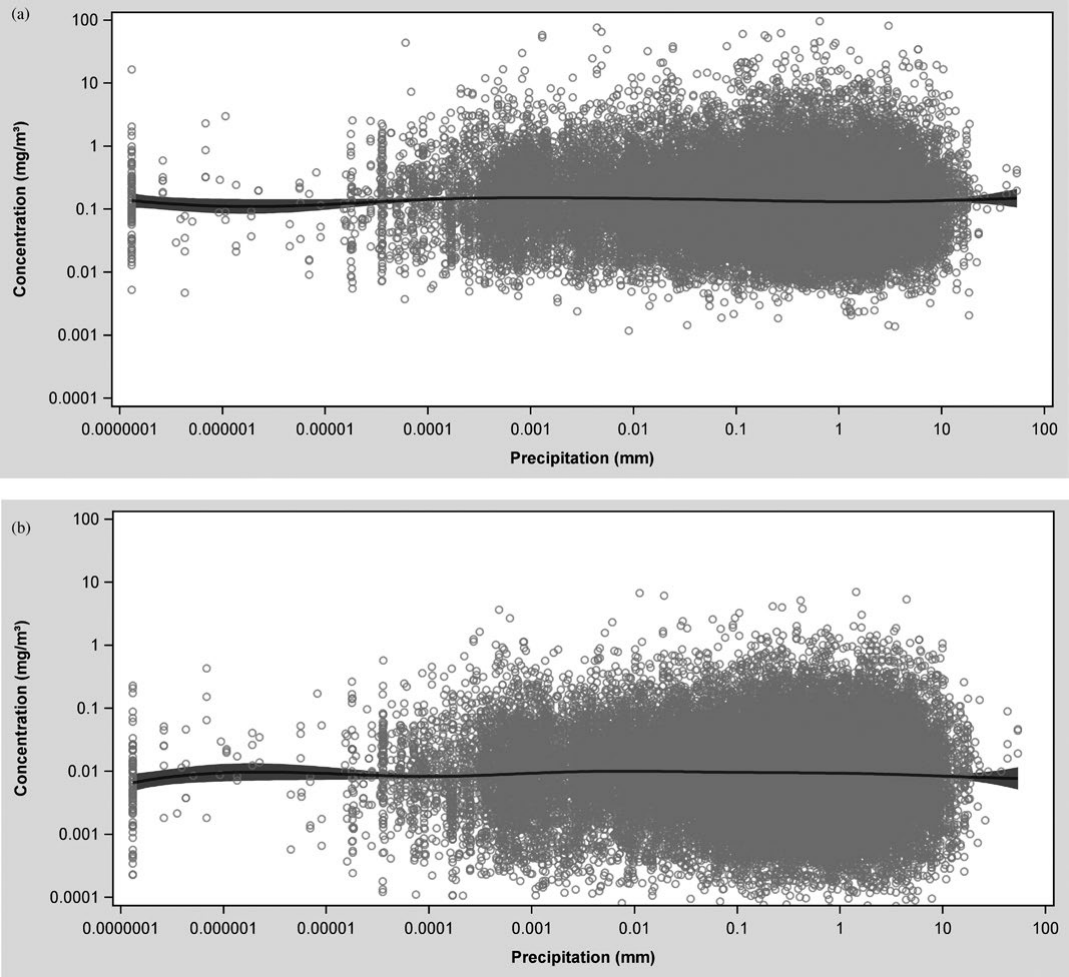
Spline plot of respirable dust (a) and quartz (b) concentration in mg/m^3^ versus precipitation in mm.

**Fig. 4.**
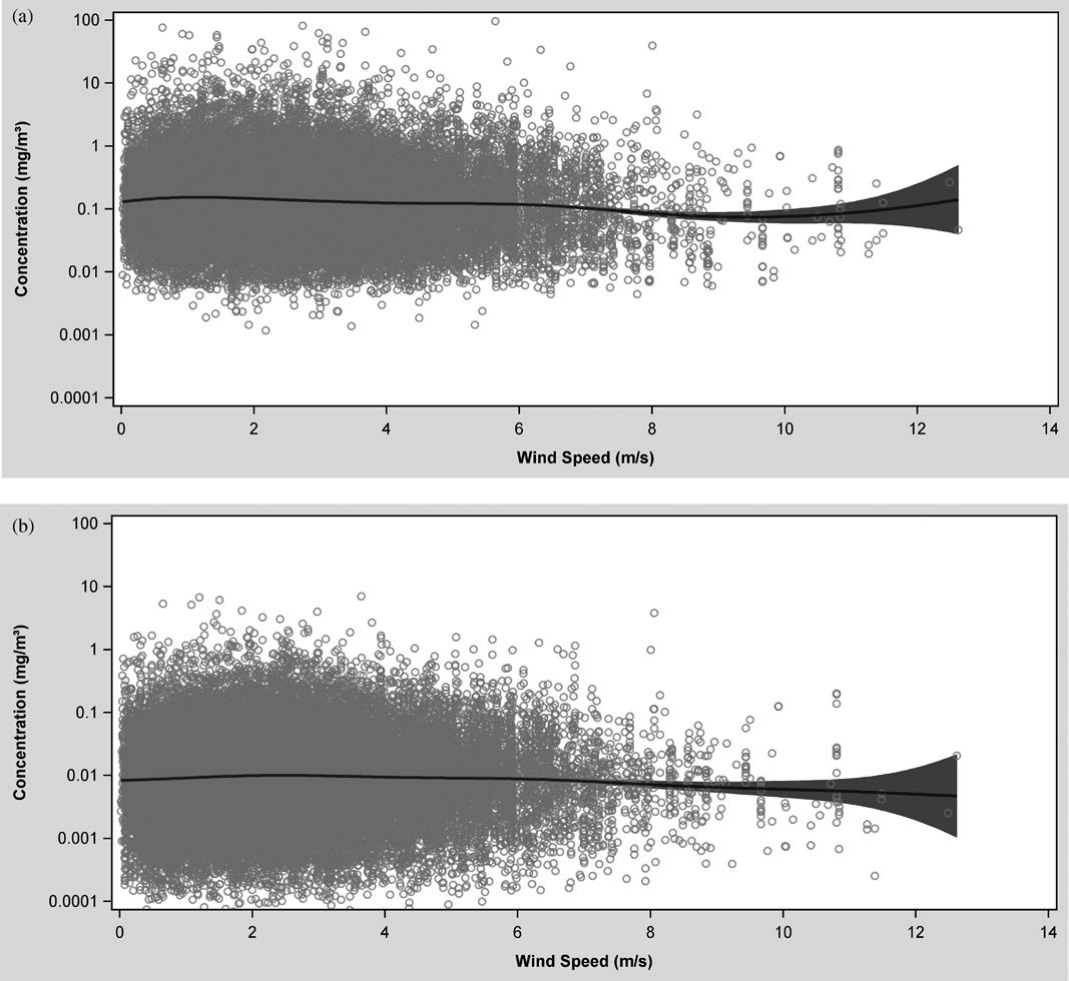
Spline plot of respirable dust (a) and quartz (b) concentration in mg/m^3^ versus wind speed in m/s.

Distinctive patterns in dust and quartz exposure concentrations were most evident in the temperature-exposure and wind speed-exposure splines (Figs 2–4). Outdoor temperature varied over a wide range (~ −20 to ~ +30 °C); however, most of the data were collected on days with outdoor temperatures between ~0 and ~25 °C. On average, the highest respirable dust and quartz concentrations were measured on days with extremely low outdoor temperatures (−10 and −12 °C); however, only a few measurements were taken at this temperature range. The sharpest drop in concentration was observed between −10 and 6 °C for both agents. At outdoor temperatures above 6 °C, respirable dust and quartz concentrations appeared to increase slightly. At around ~20 °C respirable dust concentrations continued to increase with a steeper gradient, while respirable quartz concentrations started to decrease (Fig. 2). At lower precipitation levels (<10 mm), no clear trend was observed in respirable dust and quartz concentrations as precipitation increased (Fig. 3). However, at higher precipitation levels (>10 mm), exposure concentrations tended to rise, although the number of measurements collected under these meteorological conditions were extremely limited. Respirable dust and quartz concentrations decreased with outdoor wind speeds up to ~10 m/s; thereafter, dust concentrations began increasing and quartz concentration began decreasing at outdoor wind speeds exceeding 10 m/s (Fig. 4).

Our linear mixed models support the hypothesis that meteorological variables can influence respirable dust and quartz concentration (Table 3). Outdoor temperature, precipitation, and wind speed were almost all significantly associated with respirable dust and quartz concentration, except for the relationship between respirable dust and wind speed, which did not reach statistical significance. When standardized across the IQRs, outdoor temperature had the strongest effect and precipitation the weakest. For every 10 °C increase in temperature and for every 10-fold increase in wind speed (e.g. from 1 to 10 m/s) and precipitation (e.g. from 1 to 10 mm), the following magnitudes of effect were observed: respirable dust; +2.5%, −1.3%, and −0.2% and respirable quartz; +6.4%, −3.3%, and −0.2%, respectively.

**Table 3.** Linear mixed model to estimate the effect of outdoor temperature, precipitation, and wind speed on respirable dust and quartz concentrations while controlling for long-term time trends, mineral produced, job performed, and site.

	Respirable dust				Respirable quartz			
Effect	β	Standard error	Effect IQR (%)	P-Value	β	Standard error	Effect IQR (%)	P-value
Intercept	-1.109	0.169		0.001	-4.173	0.161		<.0001
Temperature (°C)	0.002	0.001	2.49	0.008	0.006	0.001	6.44	<.0001
Log_10_ Precipitation (mm)	-0.026	0.005	-0.20	<.0001	-0.032	0.006	-0.24	<.0001
Log_10_ Wind speed (m/s)	-0.036	0.020	-1.26	0.065	-0.094	0.024	-3.27	0.0001
Time trend	-0.020	0.002		<.0001	-0.027	0.002		<.0001
Early time period	1.101	0.069		<.0001	0.038	0.086		0.6604
Middle time period	0.249	0.124		0.045	-0.519	0.154		0.0008
Early time period * Campaign (4-41)	-0.071	0.004		<.0001	-0.019	0.005		<.0001
Middle time period * Campaign (4-41)	-0.003	0.005		0.603	0.015	0.007		0.0244
Random effects	β (β*)				β (β*)			
Mineral	0.13(0.18)				0.09(0.08)			
Site (within mineral)	0.48(0.60)				0.68(0.74)			
Job title (within site)	0.32(0.34)				0.43(0.44)			

This translates as follows: a respirable dust sample with a concentration of 0.50 mg/m³ taken at 6.7 °C would have an estimated concentration of 0.51 mg/m³ at 17.6 °C. Similarly, a quartz sample with a concentration of 0.30 µg/m³ would be estimated as 0.32 µg/m³ over the same temperature range. A respirable dust sample with a concentration of 0.50 mg/m³ taken at an outdoor wind speed of 1.14 m/s would be estimated as 0.49 mg/m³ at 3.4 m/s, while a quartz concentration of 30 µg/m³ would be estimated as 29 µg/m³. Although these differences are statistically significant, their effects are relatively low (<10%). For precipitation, no noticeable change would occur over the IQR of 0.03 to 1.22 mm.

The sensitivity analyses produced model estimates for 10-fold increase meteorological variables that were mostly similar to that of the main model; however, there were some differences (Table S1). In the main model, we estimated a +2.22% increase in dust concentrations as temperature increases. In the sensitivity analysis, we estimated a larger increase in concentration as temperature increases (+4.89%) for measurements taken in southern region. However, we estimated a decrease in dust concentration as temperature increases for measurements with concentrations above the LoD (−2.91%) and those taken at (low) temperatures ≤6 °C (−14.1%). In the main model, we estimated a −2.57% decrease in dust concentrations as precipitation increases, and in the sensitivity analysis, we estimated a larger decrease for measurements taken in the eastern region (−6.42%). In the main model, we estimated a −3.54% decrease in dust concentrations as wind speed increases, whereas in the sensitivity analysis, we estimated a larger decrease for measurements taken at higher temperatures and in the western region (−8.51% and −6.27%, respectively). In the main model, we estimated a +5.87% increase in quartz concentrations as temperature increases, and this increase was more pronounced for measurements with temperatures >6 °C (+9.15%). In the main model, we estimated a −8.97% decrease in quartz concentrations as wind speed increases; again, this decrease was larger for measurements taken in the western region (−14.1%).

## Discussion

There are multiple factors that influence respirable dust and quartz concentrations. Our descriptive analysis investigating the relationship between outdoor meteorological conditions and sampling strategy showed similar temperature, precipitation, and wind speed on measurement and non-measurement days for participating sites of the IMA-DMP. This suggests that, relative to these meteorological conditions, measurement days are representative of all days within the participation period of the IMA-DMP sites. Furthermore, when these meteorological variables were included in the determinants of exposure linear mixed-effects model, they showed very small but statistically significant associations with respirable dust and quartz concentrations. Temperature appeared to be the most important meteorological determinant for both respirable dust and quartz, followed by windspeed, and precipitation had the least effect. All 3 meteorological conditions produced larger effects on respirable quartz concentrations than respirable dust concentrations.

It is common for the exposure-measurement strategies employed by companies to face scrutiny or for industries to be accused of preferentially sampling on days or at times when exposures are expected to be lower. A classic historical case of the latter is in recurring finding of tampering within the United States Mines’ dust monitoring program, which led to the conclusion that industries should not monitor themselves (Boden and Gold 1984; Weeks 1991, 2003). These historical situations evoked distrust in organisations’ ability to collect representative exposure samples and independently monitor exposures without introducing favourable biases. The results of our study suggest that participating companies of the IMA-DMP did not systematically sampled on days with “more favourable weather” conditions. Therefore, we anticipate no biases in the measurement strategy of the IMA-DMP participants due to meteorological conditions.

The results of our model showed that as outdoor temperature increases, respirable dust and quartz concentrations also slightly increase, whereas increases in precipitation and wind speed were associated with a small decrease in concentration of both respirable dust and quartz. In practical terms, this suggests that we expect lower exposure concentrations on cooler days with higher precipitation and higher winds. Measuring concentrations on days with higher daily temperatures would result in a very slight overestimation of long-term respirable dust and quartz concentrations by ~10 µg/m³ for respirable dust and 2 µg/m³ for respirable quartz. A reduction of similar magnitude was observed for consistently measuring concentrations on days with higher wind speed. While we did not find other studies investigating personal respirable exposure measurements and meteorological conditions in an occupational setting, we did find ambient air studies investigating these relationships. Ediagbonya and colleagues conducted an ambient air study measuring meteorological conditions (wind speed, relative humidity, and temperature) multiple times per week during respirable dust sampling (Ediagbonya et al. 2013). They examined the relationship between respirable dust concentration and meteorological variables and found a positive correlation between respirable dust and temperature (*r* = 0.71), while negative correlations were observed between respirable dust and both wind speed (*r* = −0.69) and relative humidity (*r* = −0.68). The authors concluded that respirable dust concentrations increase with rising temperature and decrease with higher relative humidity and wind speed (Ediagbonya *et al.* 2013). Sahu and colleagues found somewhat different results, they found a negative correlation between area respirable dust and temperature and wind speed (Sahu *et al.* 2018). On the contrary, Duarte and colleagues in a scoping review suggested that as wind speed increases, dust concentrations also increase but concentrations decreased due to wet deposition (Duarte *et al.* 2022). Our sensitivity analyses showed that the effects of meteorological conditions might be different under specific conditions or in certain regions. However, the absolute effects in term of increased or decreased concentrations were still small (<12%).

Multiple factors can impact the relationships between respirable dust and quartz. Hotter days tend to be drier, allowing airborne particulate matter, particularly smaller particles with lower aerodynamic diameters, to remain suspended longer, thereby leading to higher measured exposure concentrations (Dbouk and Drikakis 2022; Merenda *et al.* 2024). This effect is believed to be more prominent in low humidity and low wind speed conditions. Additionally, increased use of natural ventilation (opening windows and doors) during warm weather may cause resuspension of settled respirable particulate matter, further elevating measured concentrations. However, it can be hypothesised that when natural ventilation, particularly “fresh” uncontaminated air, is introduced especially during periods of higher wind speeds, the higher air exchange rate may lead to an overall decrease in concentrations, thereby offsetting the increase in concentration caused by resuspension. Wind speed can have both positive and negative effects depending on several factors such as the outdoor dust concentration, penetration behaviour of particulate matter, wind speed, and more importantly wind direction (Shilton *et al.* 2002; Cichowicz *et al.* 2020). A decrease or increase in outdoor exposure concentration due to wind speed has been explained by the mechanical air exchange that occurs, moving dust from one location to another thereby increasing or decreasing concentration in the measured environment. In contrast, moisture in the air can promote the hygroscopic growth of dust, especially when concentrations are low, resulting in wet deposition and lower concentrations (Hänel 1981; Pankow 2010; Zhao *et al.* 2020; Duarte *et al.* 2022). In general, meteorological conditions had a greater influence on quartz concentrations than on dust concentrations. This might be related to the fact that respirable quartz is expected to originate from a few specific sources (mining activities) and therefore has a narrower particle size distribution, while respirable dust will originate from more sources and hence will have a wider particle size distribution (Huggins and Meyers 1986; Chubb and Cauda 2017).

The IMA-DMP has systematically and routinely collected personal respirable dust and quartz samples since the early 2000s and represents hundreds of sites across Europe. Similarly, reliable point estimates of meteorological conditions can be readily extracted from the ERA5-LAND database over several decades from 1940 onwards. The characteristics of both databases allowed us to precisely study the relationship between exposure and outdoor meteorological conditions over time. Nevertheless, these databases have some limitations. The measurements in the IMA-DMP database represent several jobs that are carried out primarily indoors, but some are outdoor jobs. Detailed information on the whereabouts of the monitored workers was not available. Additionally, the meteorological data from ERA5-LAND have relatively coarse resolution and were linked to sites based on a single geocode, often representing large surface areas. Using meteorological data collected with portable sensors would have been preferable, but not available. Local weather station data would have been an alternative; however, our study involved a vast number of sites, of which many without local weather stations. Therefore, the use of a validated meteorological database such as ERA5-LAND-Land was a valid alternative (Muñoz Sabater, 2019; Blanco et al. 2023; Gomis-Cebolla *et al.*, 2023). Despite these limitations, we found statistically significant associations between respirable dust and quartz and the meteorological variables studied. These associations are however small, indicating that other factors are more relevant for personal respirable dust and quartz concentrations. Therefore, further studies are required to understand whether the positive and negative associations we observed are due to the direct effect of outdoor meteorological conditions on exposure of outdoor workers or affecting ventilation conditions of indoor workers. It is also possible that the observed associations between outdoor meteorological conditions and exposure levels are influenced by changes in work patterns or task assignments that occur in response to weather conditions (for example, reduced outdoor activity during extreme rainfall or heat). Future studies could incorporate meteorological measurements collected in situ using portable sensors worn by workers alongside detailed contextual information on sampling conditions and work activities.

Thus, our study found no evidence of companies preferentially sampling on days with more favourable outdoor weather conditions. We found very small but statistically significant associations between outdoor meteorological conditions and respirable dust and quartz concentrations, with stronger effects observed for respirable quartz. As temperature increases above (particularly above 6 °C), respirable dust and quartz concentrations tend to rise, while increases in precipitation and wind speed lead to lower concentrations. These effects remain relatively small and are consistently below 10% of measured concentrations. However, understanding these influences is essential for the developing unbiased measurement strategies for respirable dust and quartz.

## Supplementary Material

wxaf060_suppl_Supplementary_Tables_S1_Figures_S1-S2

## Data Availability

The data supporting the findings of this study are not publicly available due to proprietary agreements protecting the privacy of individuals and organisations involved. De-identified data may be shared upon reasonable request, subject to approval by the data owners (IMA-Europe).
