# Supplementary material for: Meteorological conditions hardly influence measurement strategy and measured respirable dust and quartz concentrations in the industrial minerals sector
Source: Ann Work Expo Health. 2025 Oct 6;70(1):wxaf060. doi: 10.1093/annweh/wxaf060 (PMC12822605; doi:10.1093/annweh/wxaf060)
Supplement: wxaf060_suppl_Supplementary_Tables_S1_Figures_S1-S2 [file wxaf060_suppl_supplementary_tables_s1_figures_s1-s2.pdf]

Meteorological conditions hardly influence measurement strategy and measured respirable dust and quartz concentrations in the industrial minerals sector

Nicola Blagrove-Hall[1*a], Remko Houba[2b], Alonso Bussalleu[3c], Hans Kromhout[1d]
[1]Institute for Risk Assessment Sciences (IRAS) Utrecht University, Utrecht, NL
[2]Netherlands Expertise Centre for Occupational Respiratory Disorders (NECORD) Utrecht, NL
[3]Swiss Tropical and Public Health Institute Allschwil, Switzerland

Table S1

Description of job functions in the IMA-DMP as per Annex 2 of the IMA-Europe standardized dust monitoring protocol, 2006 (Aurbutin & Meunier, 2006).

General job category	Example of tasks/activities description
Quarry operator (outdoor)	Works in quarry<br>Load dumper using an excavator Feed the crusher in quarry<br>Transport raw materials to the unloading places using a dumper or wheel loader
Crusher operator (indoor)	Feed the crusher in plant Control of crusher in plant
Wet process operator	Supervise the process in a control room Sampling and control of sieve
Dry process operator	Supervise the process in a control room Sampling and control of sieve
Miller operator	Supervise the process near the mill
Bagging operator	Supervise automatic bagging machines and bulk loading into 25/50kg bags<br>Add bag to semi-automatic bagging machine<br>Fill powder bags and cover pallets with plastic films Handle bags on pallets
Transport/bulk loading	Stock the product in the storage building<br>Fill the hopper with end product using a wheel loader Supervise the conveyor belt feeding ship/train/truck<br>Load goods in lorries and organize the storage operation
Foreman/plant management staff	General office work<br>Supervise and organize plant activities
Maintenance	Control of process and product<br>Control of plates in the crusher, new sieves, dust sealing, checking and cleaning inside enclosures<br>In charge of mechanical and electrical maintenance in plant/office/quarry
Multi-skilled*	Does several job functions, none of which amounting to or exceeding 50 % of his working time
Laboratory workers	Samples collection in the plant, analysis & quality control of the samples
Plastification	Manufacture prepared body from clay

* For this category, it is necessary to fully describe the activities of the workers as well as the time percentages for each activity.

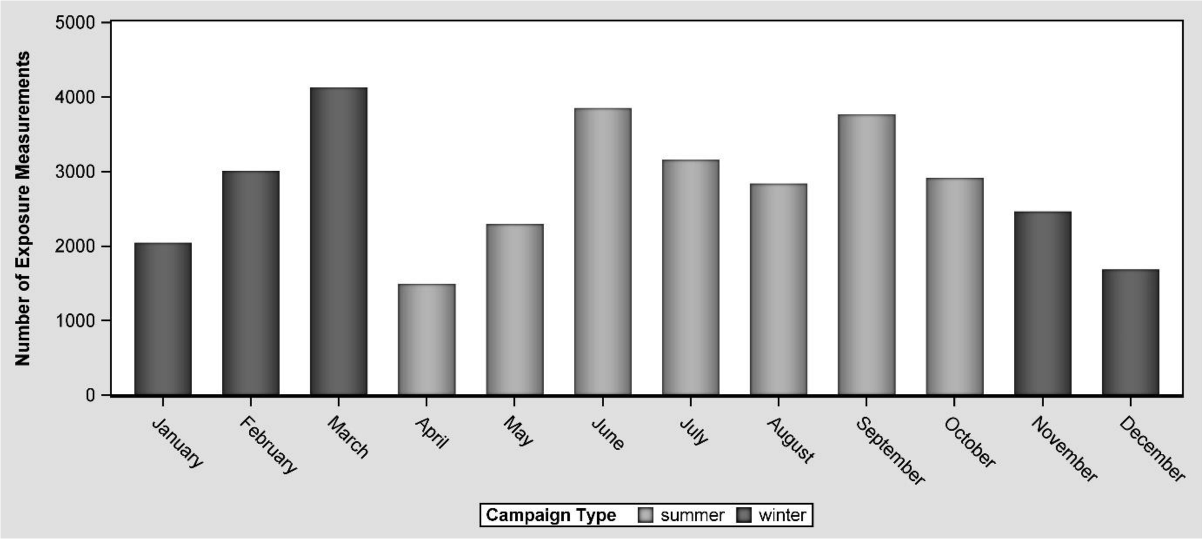

Figure S1. Number of samples collected per month by sampling campaigns.

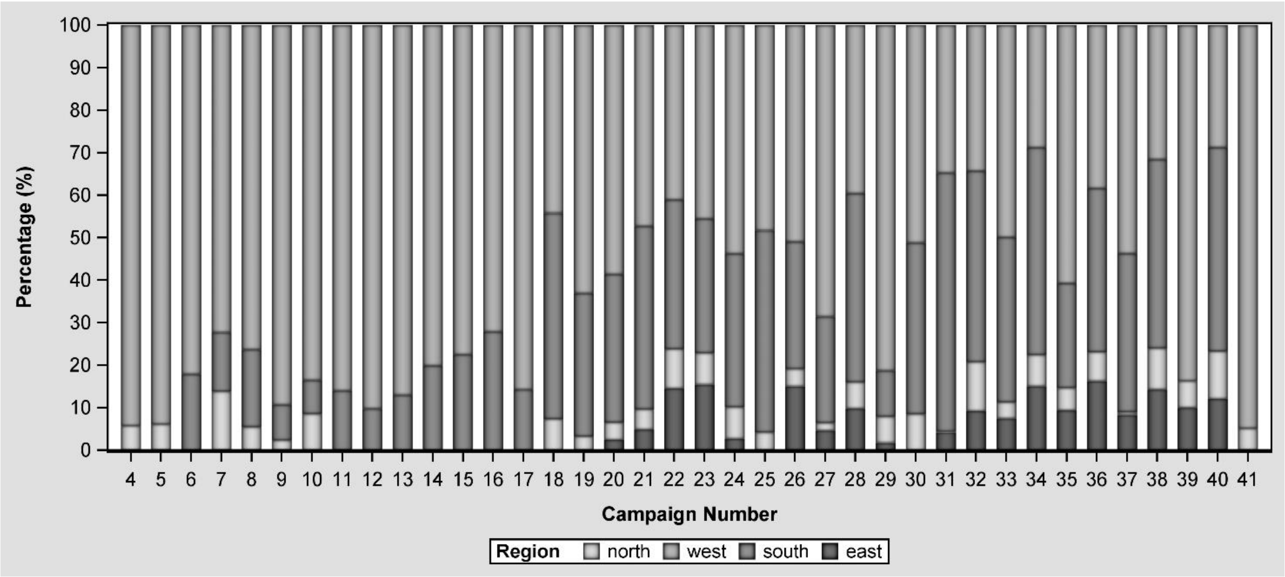

Figure S2. Annual distribution of samples by region over the sampling campaigns of the IMA-DMP.

Table S2

Sensitivity Analyses. Mixed effects linear regression model results of the restricted database (without imputed measurements below LoD) and the stratified database (i.e., by temperature ≤ 6 ⁰C and > 6 ⁰C and ii. by region).

		Respirable dust				Respirable quartz			
		β	Standard error	Effect	P-Value	β	Standard error	Effect	P-Value
Full data set n= 33,679	Intercept	-1.11	0.17		0.001	-4.17	0.16		<.0001
	Temperature (°C)	0.002	0.001	2.22*	0.01	0.01	0.0001	5.87*	<.0001
	Precipitation (mm)	-0.03	0.005	-2.57**	<.0001	-0.03	0.01	-3.15**	<.0001
	Wind speed (m/s)	-0.04	0.02	-3.54**	0.07	-0.09	0.02	-8.97**	0.0001
Measurements above LoD, n= 18,785	Intercept	-0.86	0.17		0.002	-3.78	0.19		0.000
	Temperature (°C)	-0.003	0.001	-2.91*	0.002	-0.0002	0.001	-0.17*	0.88
	Precipitation (mm)	-0.02	0.01	-1.71**	0.001	-0.02	0.01	-1.79**	0.003
	Wind speed (m/s)	-0.04	0.02	-3.74**	0.09	-0.09	0.03	-8.69**	0.0004
High temperature > 6 ⁰C, n= 26,364	Intercept	-1.26	0.16		0.0003	-4.24	0.17		<.0001
	Temperature (°C)	0.003	0.001	3.51*	0.01	0.01	0.002	9.15*	<.0001
	Precipitation (mm)	-0.03	0.01	-2.73**	<.0001	-0.04	0.01	-3.69**	<.0001
	Wind speed (m/s)	-0.02	0.02	-2.12**	0.34	-0.07	0.03	-6.35**	0.02
Low temperature ≤ 6 ⁰C, n= 7,315	Intercept	-0.73	0.26		0.03	-3.57	0.25		<.0001
	Temperature (°C)	-0.02	0.004	-14.1*	0.0002	-0.002	0.005	-1.85*	0.71
	Precipitation (mm)	0.005	0.01	0.50**	0.67	0.01	0.01	0.94**	0.51
	Wind speed (m/s)	-0.09	0.04	-8.51**	0.04	-0.05	0.05	-4.62**	0.38
Region- east, n= 2,131	Intercept	-1.40	0.47		0.03	-3.32	0.37		0.0003
	Temperature (°C)	0.002	0.003	2.13*	0.46	0.01	0.003	9.49*	0.01
	Precipitation (mm)	-0.07	0.02	-6.42**	0.0004	-0.04	0.02	-4.00**	0.06
	Wind speed (m/s)	0.10	0.09	10.1**	0.31	0.19	0.11	21.5**	0.08
Region- west, n= 19,006	Intercept	-1.72	0.25		0.0004	-4.26	0.20		<.0001
	Temperature (°C)	0.003	0.001	2.70*	0.03	0.004	0.002	4.21*	0.01
	Precipitation (mm)	-0.04	0.01	-3.46**	<.0001	-0.04	0.01	-3.67**	<.0001
	Wind speed (m/s)	-0.06	0.03	-6.27**	0.02	-0.15	0.04	-14.1**	<.0001
Region- north, n= 1,896	Intercept	-0.84	0.31		0.05	-1.85	0.31		0.004
	Temperature (°C)	0.002	0.004	1.95*	0.64	0.0005	0.004	0.47*	0.92
	Precipitation (mm)	0.01	0.02	0.66**	0.76	-0.02	0.02	-2.13**	0.35
	Wind speed (m/s)	-0.04	0.10	-3.94**	0.68	-0.002	0.11	-0.21**	0.98
Rgion- south, n= 19,646	Intercept	-0.35	0.16		0.10	-4.44	0.19		<.0001
	Temperature (°C)	0.005	0.001	4.89*	0.0002	0.01	0.002	7.65*	<.0001
	Precipitation (mm)	-0.003	0.01	-0.28**	0.67	-0.02	0.01	-2.13**	0.01
	Wind speed (m/s)	-0.01	0.03	-0.61**	0.83	-0.06	0.04	-5.47**	0.12

* %change per 10 ⁰C
** % change 10-fold increase
